# Peritonsillar abscess caused by *Prevotella bivia* during home quarantine for coronavirus disease 2019: Case report

**DOI:** 10.1097/MD.0000000000029469

**Published:** 2022-05-27

**Authors:** Toshinobu Yamagishi, Naoki Arakawa, Sho Toyoguchi, Koshi Mizuno, Yusuke Asami, Yurika Yamanaka, Hiroki Yamamoto, Ken Tsuboi

**Affiliations:** a Department of Emergency and Internal Medicine, Saitama Citizens Medical Center, Saitama, Japan; b Division of General Internal Medicine, Jichi Medical University Hospital, Tochigi, Japan; c Department of Otolaryngology, Saitama Citizens Medical Center, Saitama, Japan.

**Keywords:** coinfection, coronavirus disease 2019, peritonsillar abscess, *Prevotella bivia*, severe acute respiratory syndrome coronavirus 2

## Abstract

**Rationale:**

Since late 2019, severe acute respiratory syndrome coronavirus 2 (SARS-CoV-2) had rapidly spread worldwide, resulting in a pandemic. Patients with coronavirus disease 2019 (COVID-19) have difficulty in visiting clinics in person during pandemic because they might be encouraged to quarantine at home with supportive care. Peritonsillar abscess rarely coexists with COVID-19; however, patients with SARS-CoV-2 infection could get co-infections or become superinfected with other microorganisms which could cause peritonsillar abscess. We herein describe a case of peritonsillar abscess caused by *Prevotella bivia* that occurred as a co-infection with COVID-19 during home quarantine.

**Patient concerns:**

A 32-year-old Asian woman who was diagnosed with COVID-19 was instructed to stay home for quarantine. Her pharyngeal discomfort worsened, and she experienced trismus and dysphagia. An emergent visiting doctor referred her to our hospital. Contrast-enhanced computed tomography showed peritonsillar abscess findings, following which we referred her to an ear, nose, throat specialist. *Prevotella bivia* was identified on needle aspiration pus culture; however, two sets of blood and throat cultures were negative.

**Diagnosis:**

A definitive diagnosis of acute COVID-19 and peritonsillar abscess due to *Prevotella bivia* was made.

**Interventions:**

An antibiotic drug, antiviral drug, and adjunctive steroid were administered intravenously.

**Outcomes:**

Her symptoms improved without the need for incision and drainage, and she was discharged on day 7.

**Conclusion:**

Patients with suspected peritonsillar abscess should be triaged and referred to ear, nose, throat specialists appropriately. Scoring systems, such as modified Liverpool peritonsillar abscess score or the guidelines criteria might be useful tools to triage patients. During the early phase of SARS-CoV-2 infection, administration of corticosteroids is not recommended. When adjunctive steroids are considered for peritonsillar abscess, prior to or simultaneous use of the antiviral agent remdesivir for COVID-19 might be recommended.

## 1 Introduction

Toward the end of 2019, an outbreak of severe acute respiratory syndrome coronavirus 2 (SARS-CoV-2) infection was reported in Wuhan, China.^[1]^ Since then, the virus has rapidly spread worldwide, resulting in a pandemic. Recently, cases of co-infection and superinfections with SARS-CoV-2 infection have been reported.^[2,3]^ According to the Centers for Disease Control and Prevention, superinfection is defined as “an infection following a previous infection, especially when caused by microorganisms that are resistant or have become resistant to the antibiotics used earlier,^[4]^” while co-infection is defined as “an infection concurrent with the initial infection.” The difference is that co-infections occur simultaneously, whereas superinfections develop after the initial infection. Patients with a co-infection or superinfection have a higher risk of mortality than those who have only SARS-CoV-2 infection^[2]^; thus, it is essential to consider whether the patient had a co-infection or had become superinfected with other microorganisms when the clinical course worsened. The most common origin of a co-infection or superinfection is the respiratory tract.^[2,3]^ We herein describe a rare case of peritonsillar abscess that occurred as co-infection with SARS-CoV-2 infection during home quarantine for coronavirus disease 2019 (COVID-19), furthermore, this case was the first instance that *Prevotella bivia* was identified, on aspiration pus culture, as the causative microorganism.

## 2 Case presentation

A 32-year-old Asian woman was transferred to our fever clinic with complaints of fever and sore throat. Her partner was diagnosed with COVID-19 on the day of the patient's symptom onset. Previously, she had no symptoms and had been in her normal state until 7 days before this presentation when chills, fatigue, muscle pain, pharyngeal discomfort, and fever started. The next day, a nasopharyngeal swab was collected and assessed for SARS-CoV-2 RNA using reverse transcription-polymerase chain reaction (RT-PCR) at a local clinic, and the result was positive. The patient was diagnosed with COVID-19 and was instructed to self-quarantine at home. Two days before admission, she complained of pharyngeal discomfort, which progressed to sore throat with pain radiating to her left ear. She could not drink owing to worsening sore throat and had dysphagia. Her fever persisted above 38.0°C for 7 days until her admission. An emergent visiting doctor referred her to our hospital. She was 157-cm tall and weighted 45 kg, and she did not have any history of medical conditions, medication, smoking, and excess alcohol consumption. She received the booster COVID-19 vaccination 3 months before this admission.

**Figure 1:**
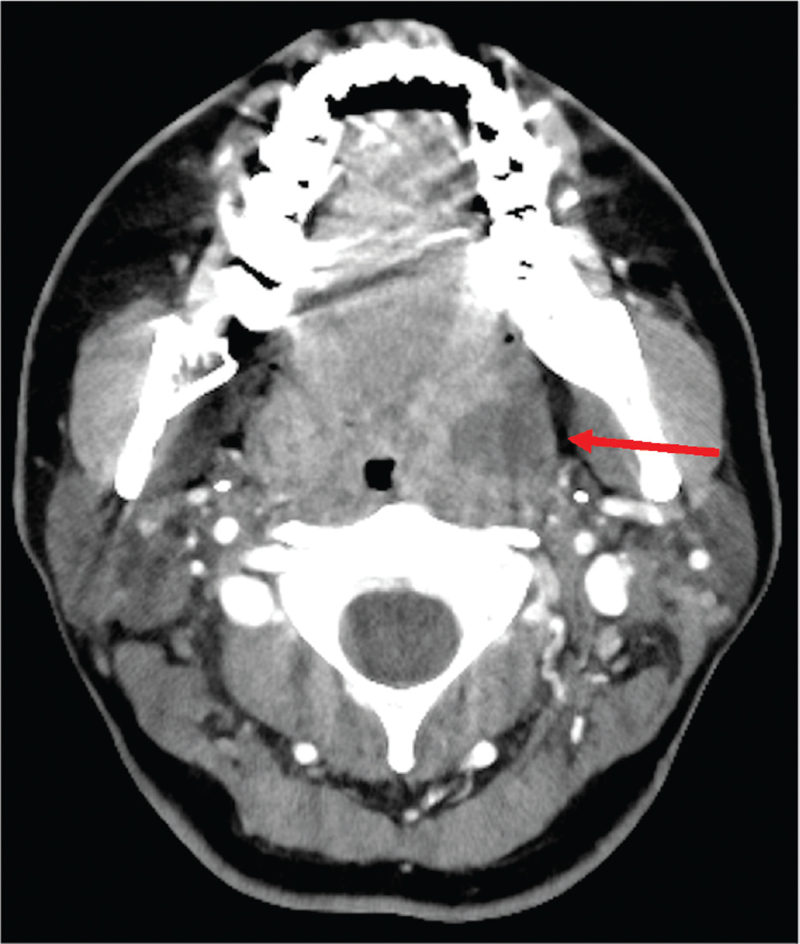
Contrast-enhanced computed tomography (CECT) in the axial view showing a low-density lesion (17.5 mm × 14.7 mm) with a ring-enhanced layer from the left-side tonsilla to the upper pharynx (red arrow).

**Figure 2:**
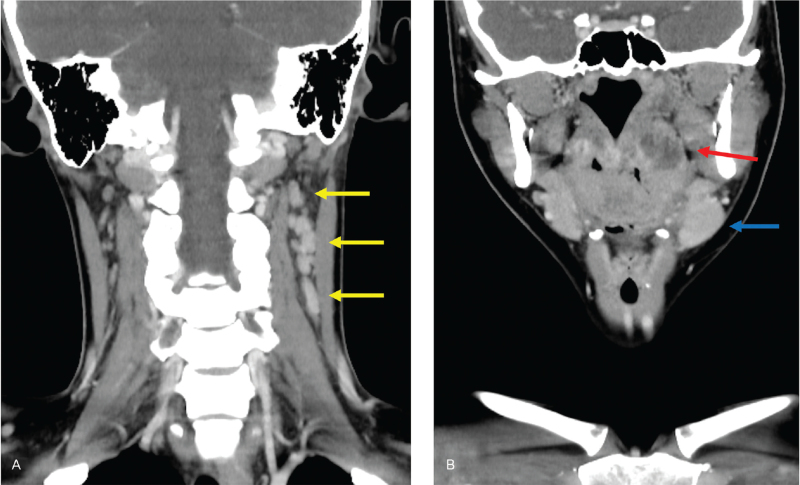
(A) Contrast-enhanced computed tomography (CECT) in the sagittal view showing left-side swollen neck lymph nodes (yellow arow). (B) CECT in the sagittal view showing a low-density lesion (19.1 mm in height) at the left-side tonsilla (red arrow) and a swollen left submandibular gland (blue arrow).

Upon arrival, the patient was awake and alert and had the following vital signs: body temperature, 36.4°C (after taking ibuprofen); respiratory rate, 18 breaths/min; blood pressure, 105/74 mm Hg; heart rate, 106 beats/min; oxygen saturation, 100% at ambient air. There was no muffled voice. She had worsened sore throat and could not open her mouth enough (only two-finger width). She had left-sided neck swelling and pain due to some enlarged lymph nodes. Her left-sided tonsil was swollen without any pus plug and deviation of the uvula. Laboratory test results were as follows: leukocyte count, 10,600 cells/μl; neutrophils, 85%; lymphocytes, 8.6%; hemoglobin, 14.8 g/dl; platelet count, 15.0 × 10^4^ cells/μl; aspartate aminotransferase, 15 U/L; alanine aminotransferase, 15 U/L, C-reactive protein, 13.4 mg/dl; procalcitonin, 0.03 ng/ml; prothrombin time international normalized ratio, 1.04; and D–dimer, 0.9 μg/ml. The other laboratory test results are shown in Table 1. Owing to suspected acute epiglottitis, tonsillar abscess, or complications such as or deep neck space infections, contrast-enhanced computed tomography (CT) was performed. CT showed a low-density lesion (19.1 mm × 17.5 mm × 14.7 mm) with a ring-enhanced layer from the left-sided tonsilla to the upper pharynx, and a swollen left submandibular gland and neck lymph nodes without any pneumonia in the lung fields (Figs. 1 and 2A,BA, B). After obtaining blood, throat, and needle aspiration pus cultures, empiric antimicrobial drug (ampicillin–sulbactam 3 g intravenously 4 times a day) was administered. We referred to an otolaryngologist, and the laryngoscopy findings showed an erythematous swollen left-sided tonsil and a left-sided lateral pharyngeal band without any pus, but with pus in the upper pharynx. There was no edematous epiglottis and bilateral arytenoids. The otolaryngologist did not perform incision and drainage because of a little amount of pus as detected on needle aspiration and the risk of SARS-CoV-2 transmission. After the administration of antiviral agents (remdesivir 200 mg on day 1, followed by 100 mg daily intravenously), adjunctive steroids (dexamethasone 3.3 mg intravenously twice a day) were also administered. A nasopharyngeal swab for RT-PCR (TaqPath SARS-CoV-2 real-time PCR Kit HT [Life Technologies Japan Co., Tokyo, Japan]) and serology test (Architect Abbott SARS-CoV-2 IgG, IgM [Abbott Japan LLC, Tokyo, Japan]) were performed to confirm acute SARS-CoV-2 infection.

**Table 1:** Laboratory data on admission and on day 4.

Variable	Reference range	Admission	Day 4
Complete blood cell count
White blood cell count (/μl)	3500–9700	10,600	7400
Differential count (%)
Neutrophils	42–74	85	68
Lymphocytes	18–50	8.6	18
Hemoglobin (g/dl)	11.2–15.2	14.8	11.4
Hematocrit (%)	34.3–45.2	43.6	34.3
Platelet count (×10^4^/μl)	14.0–37.9	15.0	20.1
Blood chemistry
Sodium (mEq/L)	135–145	139	139
Pottasium (mEq/L)	3.5–-.0	4.0	3.7
Chloride (mEq/L)	98–108	98	104
Urea nitrogen (mg/dl)	8–20	13.0	11.4
Creatinine (mg/dl)	0.46–0.82	0.59	0.61
Glucose (mg/dl)	70–109	125	101
Hemoglobin A1c (%)	4.6–6.2	5.3	
Alanine aminotransferase (U/L)	5–45	15	20
Aspartate aminotransferase (U/L)	10–40	15	16
Alkaline phosphatase (U/L)	38–113	68	43
Creatinine kinase (U/L)	50–210	40	17
Lactate dehydrogenase (U/L)	120–245	188	134
Ferritin (ng/ml)	5–157	278.6	189.9
Interferon-λ3 (pg/ml)	0–13.5	<3.0	<3.0
C-reactive protein (mg/dl)	0–0.3	13.41	3.37
Procalcitonin (ng/ml)	0–0.04	0.03	0.02
Blood coagulation
Prothrombin time international normalized ratio	0.9–1.13	1.04	1.39
Activated partial thrombin time (s)	26.9–38.1	32.2	43.7
D-dimer (μg/ml)	0–1	0.9	0.8

On the second day of hospitalization, the sore throat improved, and she could open her mouth at a three-finger width. Laryngoscopy findings showed improvement in the focal inflammation of the left-sided tonsil, left lateral pharyngeal band, and upper pharynx. Intravenous dexamethasone was discontinued on day 2. Oral intake was started on day 3. Her body temperature reached normal without antipyretics. On the fourth day of hospitalization, nausea and vomiting had persisted since the previous day, but sore throat did not worsen, and her abdominal findings and laboratory data on that day were unremarkable (Table 1). Because drug side effects were suspected, remdesivir was discontinued on that day. Her respiratory status became stable during her hospital stay, and her nausea and appetite became normal. *Prevotella bivia* was identified on aspiration pus culture; however, two sets of blood and throat cultures were negative. The results of minimum inhibitory concentrations of the antimicrobial and interpretation of susceptibility testing are shown in Table 2. Her RT-PCR result was positive, with a negative serologic test for both COVID-19 IgM and IgG. A definitive diagnosis of COVID-19 and peritonsillar abscess due to *P bivia* was made. On day 6, laryngoscopy showed further improved focal inflammation findings; thus, intravenous Ampicillin–sulbactam was switched to oral antibiotics (amoxicillin 500 mg–clavulanate 125 mg [AMPC/CLV]) three times a day, and she was discharged from our hospital without any sequelae on day 7. AMPC/CLA was prescribed for 12 days until her pharyngeal discomfort disappeared. It was then confirmed that her symptoms did not become exacerbated on an outpatient basis.

**Table 2:** Susceptibility testing for *Prevotella bivia*.

Antimicrobial agents	MIC (mg/L) and interpretation of susceptibility
Penicillin G	8 (R)
Ampicillin	>8 (R)
Cefaclor	>32 (R)
Cefazolin	16 (S)
Cefmetazole	16 (S)
Cefdinir	>4 (R)
Cefotiam	>32 (R)
Cefotaxime	8 (S)
Ceftriaxone	16 (S)
Cefcapene	>4 (R)
Cefepime	>32 (R)
Flomoxef	≤8 (S)
Imipenem-cilastatin	≤0.5 (S)
Meropenem	≤0.25 (S)
Amoxicillin-clavulanic acid	2 (S)
Ampicillin-sulbactam	≤4 (S)
Piperacillin-tazobactam	≤16 (S)
Clindamycin	≤0.25 (S)
Minocycline	8 (I)

I = intermediate, MIC = minimum inhibitory concentration, R = resistant, S = susceptible.

## 3 Discussion

This case highlighted three important clinical findings. First, patients with SARS-CoV-2 could be co-infected or superinfected with other microorganisms and develop peritonsillar abscess as seen in our case. Second, peritonsillar abscess sometimes need surgical intervention; thus, physicians should triage and refer the affected patients to an ear, nose, throat (ENT) specialist appropriately during the pandemic. Third, when we decided to use adjunctive steroids for peritonsillar abscess, we should identify the stages of COVID-19 illness, and whether or not the current phase is a viral response phase.

Co-infection of peritonsillar abscess with COVID-19 is rare. To the best of our knowledge, only one case has been reported.^[5]^ In the previous case, the causative microorganisms of abscess could not be detected. However, in our case, *P bivia*, which was resistant to penicillin G, ampicillin, and some cephalosporins in susceptibility testing, could be identified (Table 2); thus, the patient could be treated appropriately by AMPC/CLV without exacerbation on an outpatient basis. In a systematic review of case rereports of *P bivia* infections, 19 cases were reported by 2021. Co-infection with other microorganisms was reported in seven cases and abscess occurred in seven of the 19 cases.^[6]^ Thus, *P bivia* is predisposed to cause co-infection and form abscess. Some researchers have envisioned three bacterial/SARS-CoV-2 co-infections scenarios: (1) secondary SARS-CoV-2 infection following bacterial infection/colonization; (2) combined viral/bacterial pneumonia; and (3) secondary bacterial “superinfection” after SARS-CoV-2 infection. SARS-CoV-2 might enhance colonization and attachment of bacteria to host tissue.^[7]^ On the contrary, bacterial infection might dampen the activation of host defense signaling, which might increase susceptibility to SARS-CoV-2 infection. The underlying mechanisms in these scenarios are context- and time-dependent.^[7]^ In our case, we could not indicate accurately which microorganisms had caused primary infection, that is, whether SARS-COV-2 or *P bivia*. However, SARS-CoV-2 infection in the present case might have been in the acute phase because she had obvious close contact with sick person, her RT-PCR showed a positive result, and her serology test was negative. In a systematic review, IgM is detected in only 23.2% by 1 week^[8]^; thus, her negative results of serological antibodies might mean that these antibodies were not produced in sufficient amounts in the early phase. Infectious mononucleosis is reported to occur as co-infected in 1.5% to 6.0% of peritonsillar abscess cases.^[9]^ Infectious mononucleosis caused by Epstein-Barr virus might change the local bacterial defense system and exacerbate bacterial colonization on the oropharyngeal mucosal membrane owing to bacterial penetration.^[10–12]^ If SARS-CoV-2 provokes the same mechanism as that of Epstein-Barr virus, then pharyngeal injury due to COVID-19 might cause co-infection with *Provotella bivia* and result in abscess formation.

Cough, fever, myalgia, and headache are common COVID-19-related symptoms, and the proportions of cases with these symptoms were 50.3% to 62.7%, 41.3% to 43.1%, 36.1% to 54.6%, 34.4% to 51.2%, respectively. Sore throat is reported to occur at a relatively lower frequency of 20% to 33.7%^[13,14]^; however, it appears to occur more commonly with the Omicron variant (72%).^[15]^ In addition to the relatively few medical resources available during the pandemic, and given the possibility that the Omicron variant has reduced intrinsic virulence and causes reduced hospitalization among COVID-19 patients,^[16,17]^ COVID-19 patients who have sore throat but without any risk factors for severe disease might be encouraged to stay home for self-quarantine with supportive care. Thus, a non–ENT specialist should be able to distinguish the patient's condition with sore throat, especially whether or not they need referral to an ENT specialist and require oral examinations. ENT United Kingdom (UK)-released guidelines identify all aerodigestive tract examinations as having a high risk of transmission and advised avoidance of all unnecessary examinations and procedures.^[18]^ Oral examinations should be restricted to patients with a history of suspected diagnosis of quinsy, severe symptoms, or inability to swallow fluids and medication.^[18]^ Modified Liverpool Peritonsillar abscess Score (LPS) (the components are unilateral sore throat, trismus, sex [male], pharyngeal voice change [hot potato voice]) is also one of the useful tools. It demonstrates a high negative predictive value of 99% and high sensitivity of 98% for diagnosing peritonsillar abscess.^[19]^ In our case, she experienced left-sided sore throat, dysphagia, and trismus. She was considered suitable for further examination because her conditions met the ENT UK criteria and her modified LPS was 5 points. We can use this scoring system or the guidelines criteria to decide which patients require in-person consultation.

When we decided to use adjunctive steroids for peritonsillar abscess to relieve pain and improve oral fluid intake (that is included in the initial treatment regimen in the ENT UK guidelines), we should estimate the stages of COVID-19 illness. In the viral response phase, that is, from early establishment of the disease to viral multiplication and localized inflammation in the lung, patients often have mild symptoms such as malaise, fever, and a dry cough.^[20]^ During the viral response phase, corticosteroids inhibit immune responses and pathogen clearance and might provoke viral replication^[21]^; hence, the use of corticosteroids should be avoided during this phase. The viral response phase is followed by the host inflammatory response phase, wherein patients develop viral pneumonia with hypoxia, and in this phase, corticosteroids are recommended.^[20,22]^ Typically, peritonsillar abscess formation takes more than 4 to 7 days.^[23]^ If a COVID-19 patient was co-infected with bacteria at the same time, then a diagnosis of peritonsillar abscess was made, and there is a possibility of being in the viral response phase. In our case, it is difficult to deduce whether or not the SARS-CoV-2 infection phase was the viral response phase; however, chest CT findings did not indicate pneumonia. Hence, at least she might not be in the host inflammatory response phase with hypoxia. Initiation of the antiviral agent remidesivir (an inhibitor of the viral RNA-dependent, RNA polymerase with in vitro inhibitory activity against SARS-CoV-2^[24]^) before or simultaneously with dexamethasone is associated with a trend towards faster viral clearance compared with initiating an antiviral agent after dexamethasone or not using remdesivir.^[25]^ In our case, remdesivir was also used with dexamethasone simultaneously owing to concerns about COVID-19 deterioration.

This case study has a limitation. The exact variant of SARS-CoV-2 in our case is unclear. The Omicron variant was identified in South Africa on November 24, 2021, for the first time; since then, the variant had rapidly spread worldwide.^[16]^ As of January 2022, the Omicron variant accounted for more than 90% of all circulating viruses in the United States.^[26]^ Similarly, In Japan, the number of COVID-19 cases skyrocketed since the beginning of January 2022 owing to the Omicron variant, which is responsible for the sixth wave, with an estimated proportion of more than 90%.^[27]^ Under this circumstance, the viral S gene was not detected in the RT-PCR test (S gene target failure) in our case; thus, the patient was likely to be infected by the Omicron variant.^[28]^

## 4 Conclusion

Co-infection of peritonsillar abscess with COVID-19 rarely occurs; however, patients with SARS-CoV-2 could be co-infected or superinfected with other microorganisms and could develop peritonsillar abscess. Patients with sore throat without dyspnea face difficulty in visiting the clinic in person during the pandemic. However, we should triage patients with suspected peritonsillar abscess who did not undergo oral examination, and we should refer such patients to an ENT specialist for further examination. Scoring systems, such as modified LPS or the guidelines criteria might be useful tools to triage patients. During the early phase of SARS-CoV-2 infection, administration of corticosteroids is not recommended. When adjunctive steroids are considered for peritonsillar abscess, prior or simultaneous use of the antiviral agent remdesivir for COVID-19 might be recommended.

## Acknowledgments

The authors would like to thank Editage (https://www.editage.jp) for their English language editing.

## Author contributions

**Conceptualization:** Toshinobu Yamagishi.

**Data curation:** Toshinobu Yamagishi and Yurika Yamanaka.

**Supervisions:** Yurika Yamanaka, Hiroki Yamamoto, and Ken Tsuboi.

**Writing – original draft:** Toshinobu Yamagishi.

**Writing – review & editing:** Naoki Arakawa, Sho Toyoguchi, Koshi Mizuno, Yusuke Asami, Yurika Yamanaka, Hiroki Yamamoto, and Ken Tsuboi.
